# Achieving Precise Spectral Analysis and Imaging Simultaneously with a Mode-Resolved Dual-Comb Interferometer

**DOI:** 10.3390/s21093166

**Published:** 2021-05-03

**Authors:** Zejiang Deng, Yang Liu, Zhiwei Zhu, Daping Luo, Chenglin Gu, Zhong Zuo, Gehui Xie, Wenxue Li

**Affiliations:** 1 State Key Laboratory of Precision Spectroscopy, East China Normal University, Shanghai 200062, China; zejiangdeng@foxmail.com (Z.D.); yliu@lps.ecnu.edu.cn (Y.L.); zhuzhiweiwb@163.com (Z.Z.); dapingluo@163.com (D.L.); clgu@lps.ecnu.edu.cn (C.G.); zhongzuo_ecnu@foxmail.com (Z.Z.); ghxie0121@163.com (G.X.); 2 Collaborative Innovation Center of Extreme Optics, Shanxi University, Taiyuan 030006, China

**Keywords:** dual-comb spectroscopy, gas absorption spectrum, imaging

## Abstract

In this paper, we report a scheme providing precise spectral analysis and surface imaging, simultaneously, based on a high-coherence dual-comb interferometer. With two tightly phase-locking frequency combs, we demonstrate a high-coherence dual-comb interferometer (DCI) covering 188 to 195 THz (1538.5 to 1595.7 nm) with comb-tooth resolution and a max spectral signal-to-noise ratio (SNR) of 159.7. The combination of the high-coherence dual-comb spectrometer and a reference arm simultaneously enables gas absorption spectroscopy and for the absolute distance information to be obtained in one measurement. As a demonstration, we measure the spectrum of CO_2_ and CO. From the same interferograms, we demonstrate that distance measurement, by time-of-flight (TOF), can be resolved with an rms precision of 0.53 μm after averaging 140 images and a measurement time of 1 s. Finally, we demonstrate that non-contact surface imaging, using 2D mechanical scanning, reaches lateral resolution of 40 μm. The longitudinal precision is 0.68 μm with a measurement time of 0.5 s. It verifies that DCS has the potential to be applied in standoff detection, environmental pollution monitors, and remote sensing.

## 1. Introduction

The optical frequency comb (OFC) has been demonstrated to be a powerful tool with many attractive features in many applications, including optical clocks [1,2], microwave generation, and ultra-precision metrology in fundamental physics [3,4,5,6,7]. The OFC presents discrete modes in the optical frequency domain. Dual-comb spectroscopy (DCS) employs the advantage of the OFC to down-conversion of the optical spectrum to the radio frequency (RF) spectrum. In general, OFCs have slight differences in repetition rate. However, the repetition of local comb is several times the signal comb in some special designs [8]. These two combs have big differences in repetition rates which improve the resolution of DCS effectively. In the frequency domain, these discrete laser modes of the OFC heterodyne with each other. The slightly different repetition rate ensures that the heterodyne interferograms between every pair of comb teeth have different frequency. Every heterodyne frequency in the RF domain is related to an optical mode with a down-conversion factor [9,10,11,12]. DCS has attracted considerable attention as a compact, rapid, and accurate approach with individual comb line measurements [13,14,15]. Compared with the conventional Fourier transform spectroscopy (FTS) [16], dual-comb spectroscopy has advantages in acquiring speed and spectral resolution without a mechanical delay stage. DCS provides complex responses, including amplitude and phase. High coherence is essential to take full advantage of DCS, which ensures stable comb teeth in the RF domain. A precise phase-locking system realizes the coherence. The dual-comb interferogram is transformed to dual-comb spectroscopy by fast Fourier transform (FFT). DCS in our system is mode-resolved on account of the high coherence between two combs.

DCS is an effective tool in absolute distance measurement when using the method of time-of-flight (TOF) [17,18] or the method of phase shift [19,20,21]. DCS enables ranging, fine spectra and hyperspectral imaging in remote sensing [22,23,24,25]. The optical frequency domain is converted to the RF domain with a multi-heterodyne interferogram between two combs by down-conversion. The time delay in the optical domain is down-converted from the picosecond scale to the microsecond scale. The interferogram with microsecond time duration can be measured by a digitizer. Furthermore, combining mechanical scanning with direct single-spot measurements is an effective and common method for non-contact surface profiling and mapping [26,27]. For imaging with a more compact and high-speed system, scan-less dual-comb microscopy has also been demonstrated using the spectrum encoding method [19] with a grating or a virtually imaged phase array (VIPA) [28]. We note that pulse counts in the time domain allow the absolute distance to be measured, and the pulse interferogram transforming into the frequency domain enables absorption spectroscopy. Therefore, the simultaneous measurement of the spectrum and the absolute distance can be realized in a single detection employing DCS. Such rich data measurement has the potential to be applied in air pollution monitoring [29,30,31] and remote sensing [32]. Our system provides a new standoff detection method to detect biological aerosols, explosives, and highly energetic materials [33,34,35]. Simultaneous ranging and spectroscopy capability improve the classification and identification of targets. It is a powerful type of technology in which dual-comb spectroscopy and imaging are combined in a single measurement, which has not been reported in previous works.

This paper presents the system to simultaneously measure the distance and the molecular gas absorption spectrum. This system has the ability to analyze the component of the sample and the shape of the sample without contact. The spectral analysis and the absolute distance information are acquired in a single measurement by utilizing a highly coherent DCS with a reference arm. The reference and signal arms are separated by a polarization beam splitter (PBS), which frees the dead zone in distance measurement. Tightly phase locking to a single-frequency continuous-wave (CW) laser ensures the high coherence between two OFCs. A 120 mHz comb linewidth is obtained with an acquisition time of 10 s. The precision of each single distance measurement is demonstrated to be 5.40 μm with a frame rate of 140 Hz, which depends on the difference in repetition rates. The distance measurement and absorption spectroscopy are extracted in the time domain and frequency domain, respectively, which do not influence each other. With 2D scanning on X and Y axes and the acquisition time of 0.5 s on each spot, the molecular absorptions spectrum and the entire surface profile of the sample are obtained. We demonstrate that the dual-comb system has a comb-tooth frequency resolution with a transform-limited linewidth of 2 Hz and a depth precision of 0.68 μm corresponding to the single sampling time in this measurement. The resolution and reliability demonstrated in our work prove that our dual-comb interferometer is an especially ideal tool for the classification and identification of samples.

## 2. Experimental Setup

**Figure 1:**
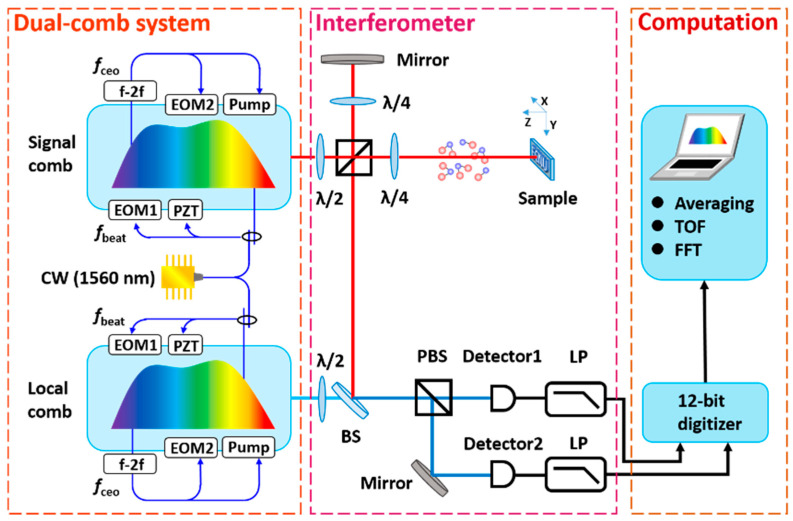
Configuration of the dual-comb interferometer to measure spectra and distance. EOM, electro-optic modulator; PZT: piezoelectric transducer; Pump: pump current; CW laser, narrow linewidth continuous-wave laser at 1560 nm; λ/2, the half-wave plate; λ/4, the quarter-wave plate; BS, the beam splitter; PBS, the polarization beam splitter; LP, the low-pass filter.

Figure 1 shows the schematic of our detection system. Two OFCs were generated from a pair of Er-doped fiber lasers by the nonlinear amplifying loop mirror (NALM) mechanism [36] for excellent noise performances. Two fiber lasers were both mode-locked with repetition rates of 100 MHz for application in DCS. All-polarization-maintaining (PM) fiber components were utilized as they are not sensitive to environmental noise [37]. Two cavities were installed in aluminum boxes to cut off the environmental disturbance, such as temperature drift and air turbulence. Every fiber cavity contains two electro-optic modulators (EOMs) for the high-speed servo control, one piezoelectric transducer (PZT) for the long-term control, and a 15 mm delay line for coarse tuning of the repetition rate. The output of each OFC was divided into three branches. The first branch was amplified, compressed, and launched into a high nonlinear fiber (HNLF) to broaden the spectrum for the carrier-envelope offset (f_ceo_) detection. The second branch was filtered by a fiber Bragg grating (FBG) and then launched into a fiber coupler with a single-frequency CW laser to detect the beat note f_beat_ between the single-frequency CW laser and the nearest comb frequency mode. FBG could improve the SNR of f_beat_. The employed single-frequency CW laser is a free-running narrow linewidth CW laser with a central wavelength of 1560 nm (OEwaves, linewidth ~10 Hz). The third branch was amplified and emitted into the optical systems in our experiment. Multiple actuators with different feedback bandwidths were utilized simultaneously to control f_ceo_ and f_beat_, respectively, which ensured the coherence of the two OFCs and the long-term stability. The high-frequency components of error signal in the feedback loop were compensated by the intra-cavity EOMs. In addition, the low-frequency components were compensated by the pump current or PZTs. The relative linewidth between the two combs is sub-hertz. In the phase-locking scheme, the two f_ceo_ signals were both stabilized at 20 MHz and the repetition rates were decided by locking f_beat_ signals at reference signals with suitable offset. Herein, the repetition rates of the two OFCs were 100, 150, 315 and 100, 150, 175 Hz. The difference in repetition rates between the two combs was 140 Hz. For synchronization of the whole system, the reference signals that were used in the phase-locking system were all referenced to an Rb atomic clock.

To simultaneously analyze the spectrum and the absolute distance in a single measurement, the output of the signal comb was divided into two orthogonally polarized beams (the signal beam and the reference beam) by a half-wave plate (HWP) and a polarization beam splitter (PBS). The signal beam was used to detect the sample. This arm contained a gas cell and a designed “ECNU” logo in a silicon slice. The gas cell was filled with 60 kPa mixed gas (50% CO_2_, 50% CO) with a transmission path of ~8 m. The letter chain was fabricated on a polished silicon substrate using a deep etching process, forming a two-step structure with the “ECNU” logo. The signal beam propagated through the gas cell. Then, it was focused on the letter chain with a diameter of 40 μm. The reference beam was reflected by a mirror at the reference arm. Two beams were overlapping again in the PBS. Meanwhile, the pulse train from the local comb propagated through the HWP and the beam splitter (BS). After using the BS, beams of two combs were combined. By rotating the HWP, the local comb beam was split into two parts and interfered with the signal and reference beams. A pair of orthogonally polarized interferograms were detected by two photodetector detectors (PD). The low-pass filters were utilized to extract the interference signals. A 12-bit data acquisition card (AT9350 Alazartech, Pointe-Claire, QC, Canada) synchronized to the Rb atomic clock was used to acquire these signals. Two interferograms were acquired by two channels with 100 MHz sampling rates and were transmitted to the computer for data processing.

## 3. Results and Discussion

**Figure 2:**
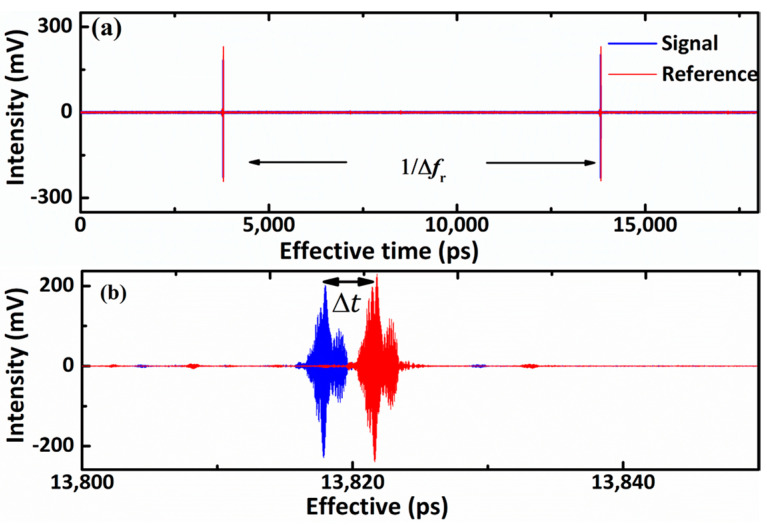
Two interferograms in the time domain. (**a**) Captured reference interferogram (red) and signal interferogram (blue). (**b**) Magnified view of interferograms.

A custom acquisition code is used to control the digitizer that allows for coherent averaging of individual interferograms to reduce the noise. For the simultaneous distance and absorption measurement, individual interferograms are recorded at ~140 Hz and averaged for 0.5 s. The signal and reference interferograms are simultaneously presented in Figure 2a. In the time domain, there is a time delay between the adjacent signal interferograms and the reference interferograms, with an exact cycle time of 1/∆*f_r_*. Moreover, the time delay between the signal and reference can be extracted from the envelop peaks of interferograms to calculate the absolute distance based on the TOF method. The time delay between DCIs and the real-time in optical has a down-conversion factor:

(1) $$\alpha =\frac{f_{rep}}{\Delta f_{rep}}$$

The magnified image in Figure 2b is used to calculate the time delay ∆*t* between two interferograms, and the absolute distance *L* is calculated by

(2) $$L=\frac{c\Delta t\Delta f_{rep}}{2f_{rep}}$$

**Figure 3:**
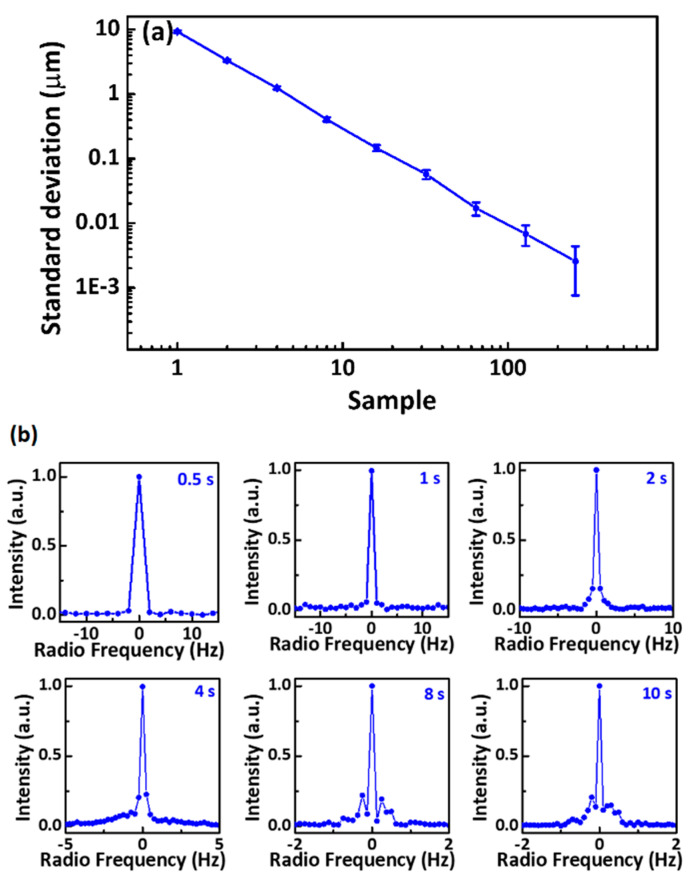
(**a**) Standard deviation of the distance measurements with different sample numbers. (**b**) The individual comb lines in the radio frequency domain directly transformed from data acquistiions with different acquisition times.

**Figure 4:**
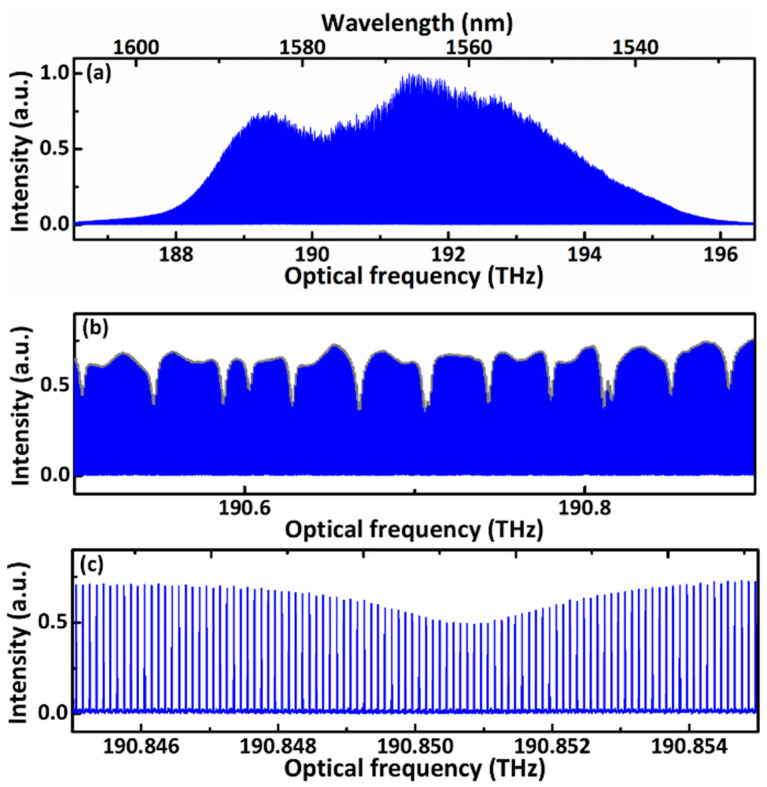
The spectrum around 193 THz with resolved comb lines. (**a**) The entire spectrum across the entire domain of emissions of the laser oscillators. (**b**) A 25× view near the absorption peaks. (**c**) A 1000× view near an absorption peak with resolved comb lines.

In the interferograms, the details contain the corresponding carriers of original pulses. Figure 2b shows the magnified view of interferograms measured from the signal and the reference arm. The envelope and carrier wave of interferograms contain the information of flighting time and spectral response, respectively. To obtain the distance and absorption from the same interferograms simultaneously, different schemes were employed to separate the information in envelope and carrier. The absorption spectrum of molecules is contained in the long tails and revivals following the central burst. The absorption information was extracted from the carrier wave by the Fourier transform to retrieve the spectral response of the gas. The temporal delay between the reference and signal interferograms is quantified instantly by measuring the peak between the envelopes. The target distance is finally worked out as the Formula (2) based on the TOF method. Based on this approach, each period of the interferograms can be used to measure the distance, as shown in Figure 3a. The corresponding absorption spectrum retrieved from the signal interferograms by FFT is shown in Figure 4.

Before scanning the sample for the results, we evaluated the precision of distance measurements and the linewidth of comb lines in the frequency domain by a series of data acquisitions. The absolute distance measurement depends on the time delay between pulses. The increase in the measurement time improves the precision. We calculated the standard deviations with the increasing averaging numbers to describe the precision of the distance measurement, and the results are plotted in Figure 3a. The slope of the deviation line is −0.502, which follows the square root trend. This behavior means that the precision is limited by white noise. With the averaging time increasing to 100, the standard deviation deviates from the square root trend limited by coherence between the two combs. The uncertainty for a single period was 5.40 μm, with the frame rate of 140 Hz. The frame rate was corresponding to the differences in the two combs’ repetition rates. The uncertainty can be improved to 0.68 and 0.53 μm with the averaging numbers of 70 and 140, respectively, corresponding to the measurement times of 0.5 and 1 s. The mode-resolved comb lines reveal the coherence of DCS and affect spectral analysis resolution. However, the transformed linewidth is limited by the acquisition time within a certain range. Figure 3b shows a series of individual comb lines directly Fourier-transformed from dual-comb interferograms with acquisition times of from 0.5 to 10 s. The transform-limited linewidths of 2 and 1 Hz with acquisition times of 0.5 to 1 s are demonstrated. When the acquisition time increased from 2 to 10 s, the linewidth of the resolved comb lines decreased. However, the line quality was degraded by the appearance of the pedestal, as shown in Figure 3b. With the increase in the measurement from 0.5 to 10 s, the limitation of the linewidth changed from acquisition times to DCS. The degradation of the linewidth is mainly caused by the noise of the free-running single-frequency CW laser, and the linewidth with an acquisition time of 10 s is demonstrated to be 120 mHz under the degradation [32]. The investigated results indicate that the dual-comb interferometer has excellent coherence in absolute distance measurement and spectral analysis.

Figure 4a displays the dual-comb spectrum ranging from 186.5 to 196.5 THz (1526.7 to 1608 nm), which was achieved by converting the RF frequency to optical frequency with an acquisition time of 0.5 s. For the observation of absorptions, the spectrum region narrowed, and the magnified view ranging from 190.5 to 190.9 THz (1571.5 to 1574.8 nm) is illustrated in Figure 4b with 20× magnification. The peaks of each comb tooth are connected with the grey line, forming the absorbed spectrum in this region. The 1000× magnification near one absorption peak around 190.851 THz (1571.9 nm) in Figure 4c contains 100 comb lines with a comb space of ~100 MHz. From the corresponding view of a single comb tooth in the RF domain, the transform-limited linewidth was 2 Hz in this case. The max signal-to-noise ratio (SNR) is 159.7. The averaging SNR from 188 to 195 THz (1538.5 to 1595.7 nm) is 91.7. The merit of our dual-comb spectra based on the averaging SNR is 6.68 × 10^6^ [8,34]. The merit of the dual-comb spectroscopy is calculated by the formula:

(3) $$Merit=\frac{SNR\times M}{\sqrt{\tau }}$$

SNR is signal to noise rate; *M* is the number of comb teeth; *τ* is the measurement time [9,38].

**Figure 5:**
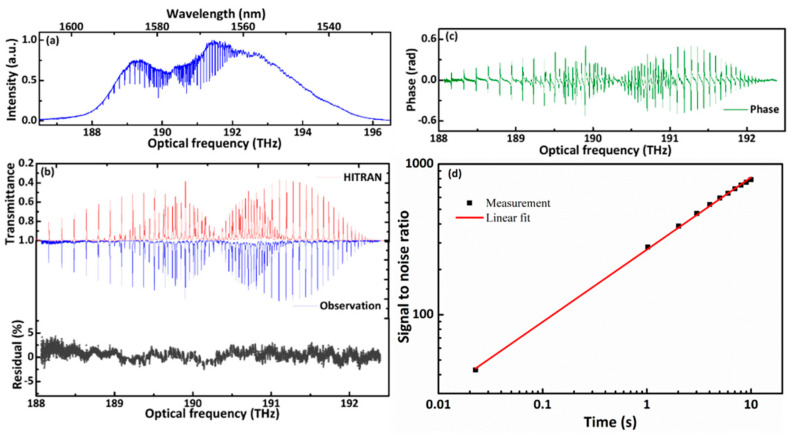
(**a**) Measured dual-comb spectrum data. (**a**) Entire spectrum from 186.5 to 196.5 THz (1526.7 to 1608.6 nm). (**b**) Normalized (blue) spectra, calculated (red) spectra, and the residual (gray) between them. (**c**) Normalized phase (green). (**d**) The evolution of the signal-to-noise ratio with measurement time.

Figure 5a shows the measured spectrum which contains the absorptions of ^12^C^16^O_2_ and ^12^C^16^O in our optical frequency region with a spectral resolution of 100 MHz. The transmission spectra cover the 30012-00001 band of ^12^C^16^O_2_ and the (3-0) band of ^12^C^16^O around 1560 nm. The transitions and phase spectra are compared with the spectrum transformed from the reference interferograms without gas absorptions in Figure 5b,c. The calculated spectrum from HITRAN is plotted opposite our experimental spectrum for comparisons in Figure 5b, and the two spectra fit well with each other. The residuals between the observations and the calculations are shown in Figure 5b. The residual of the absorption spectra is correlated to the SNR. When the SNR is high, the residual is low. The signal-to-noise is improved with increasing measurement time, as shown in Figure 5d. The maximum SNR increased from 43 to 788 with the averaging time increasing from 5.6 ms to 10 s. The slope is 0.48 with a linear fit, which indicates that the SNR is proportional to the square root of time. The slope is less than 0.5 with the free-running single-frequency CW laser. The central wavelength of the single-frequency CW laser drifts slightly with time. The accumulated frequency drift over time causes the decrease in the slope.

**Figure 6:**
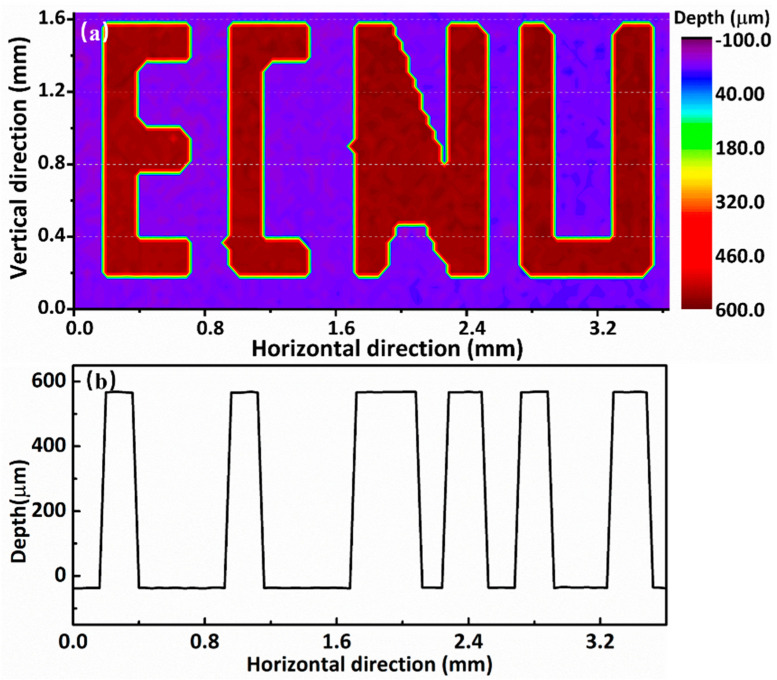
Surface mapping of the letter chain with the depth information calculated after 2D scanning. (**a**) The image of the ‘ECNU’ logo; (**b**) The depth line at 1.2 mm of vertical direction.

Moreover, we also demonstrated a time-of-flight 3D imaging that realized point-by-point imaging using this system. To image the sample by 2D scanning, we fixed the letter chain on an electric moving stage with a minimum displacement of 1 μm. According to the results in Figure 3, the acquisition time of every single spot is 0.5 s for balancing of the precision and measurement time. A smaller step would improve the lateral resolution, but it would increase the measurement time. We performed scanning on the X and Y axes with a range of 3.6 mm and 1.6 mm, respectively. The size of the picture was 90 × 60. The scanning step was set as 40 μm, because the beam diameter was also 40 μm. For the non-contact surface imaging of the sample, the absolute distance was calculated and presented on the X–Y plane, as shown in Figure 6a. The imaging is 3D map The “ECNU” logo was the hollow in a silicon wafer. Calculated by the TOF method, the height difference was 600 μm with a depth precision of 0.68 μm. Figure 6b shows the variation of depth at 1.2 mm of vertical direction. The result of depth corresponds to the thickness of the silicon wafer. The lateral resolution was 40 μm on the X–Y plane, which was limited by the signal beam diameter. Optimizing the objective lens used in the system for a smaller beam diameter can improve the lateral resolution. Depending on the scanning range, the number of image pixels was 3600, with an acquisition time of 30 min. The long-term phase stabilization of our dual-comb system ensures continuous measurement.

## 4. Conclusions

In conclusion, we have demonstrated an approach for precise spectral analysis and non-contact surface imaging. With the high-coherence DCS and a reference arm, the transmission spectrum and the absolute distance information are simultaneously obtained in one single measurement. The precision of distance measurement is 5.40 μm with a frame rate of 140 Hz. With 2D scanning, we analyzed the 3D profile and the molecular absorptions at the same time. With the acquisition time of 0.5 s for each spot, we successfully acquire the “ECNU” logo and the absorption spectra of the mixed gas. Our DCS system benefits from the tight and stable phase-locking scheme. The linewidth of the comb tooth was 2 Hz. The depth precision was 0.68 μm with a measurement time of 0.5 s. This mode-resolved dual-comb interferometer has the potential for high-resolution spectroscopy and microscopy.

According to our results, the comb line quality was degraded by the pedestal with the accumulated increases in time. This trend indicates the limit of the coherence time caused by the free-running optical reference. In future research, enhanced stability of the reference CW laser will be required to improve the comb lines and the accuracy of measurements. Furthermore, the speed of 3D mapping is limited by the 2D scanning method. A line-scan spectrum-encoded method [18] is taken into consideration for decreasing the measurement time. The improvement has a more stringent requirement regarding the optical path design while extracting the spectral absorptions encoded by the surface. However, we are confident that this scheme is attractive and has the potential for standoff detection, meeting the demand for non-contact surface mapping and inferring the chemical components with absorption spectra.

## Data Availability

Not applicable.
